# Drug-Diagnostics Co-Development in Oncology

**DOI:** 10.3389/fonc.2013.00315

**Published:** 2013-12-23

**Authors:** Richard Simon

**Affiliations:** ^1^Biometric Research Branch, National Cancer Institute, Bethesda, MD, USA

**Keywords:** predictive biomarker, clinical trial design, adaptive design, companion diagnostic, enrichment trial

## Abstract

Developments in genomics are providing a biological basis for the heterogeneity of clinical course and response to treatment that have long been apparent to clinicians. The ability to molecularly characterize human diseases presents new opportunities to develop more effective treatments and new challenges for the design and analysis of clinical trials. In oncology, treatment of broad populations with regimens that benefit a minority of patients is less economically sustainable with expensive molecularly targeted therapeutics. The established molecular heterogeneity of human diseases requires the development of new paradigms for the design and analysis of randomized clinical trials as a reliable basis for predictive medicine. We review prospective designs for the development of new therapeutics and predictive biomarkers to inform their use. We cover designs for a wide range of settings. At one extreme is the development of a new drug with a single candidate biomarker and strong biological evidence that marker negative patients are unlikely to benefit from the new drug. At the other extreme are phase III clinical trials involving both genome-wide discovery of a predictive classifier and internal validation of that classifier. We have outlined a prediction based approach to the analysis of randomized clinical trials that both preserves the type I error and provides a reliable internally validated basis for predicting which patients are most likely or unlikely to benefit from a new regimen.

## Introduction

This dominant paradigm for oncology drug development has been rapidly changing. The paradigm for development of cytotoxics involved large phase III clinical trials to find relatively small, but statistically significant, average treatment effects for target populations defined in terms of primary site and stage. The primary analysis was relatively simple, consisting of a single statistical test of the null hypothesis of no average treatment effect for the intent to treat population with regard to a single primary endpoint. Any claim of treatment benefit based on subset analysis without an overall statistically significant intent to treat analysis was viewed with suspicion.

Randomized clinical trials have made important contributions to modern medicine and public health, but they have also led to the over-treatment of broad populations of patients, most of whom don’t benefit from the increasingly expensive drugs and procedures shown to have statistically significant average treatment effects in increasingly large clinical trials. With the recognition of the molecular heterogeneity of cancer and the development of molecularly targeted drugs whose effects depend strongly on the genomic alterations and genetic background of the tumor, the broad eligibility primary site oriented clinical trial is playing a less dominant role. Increasingly sophisticated and cost effective biotechnology platforms are providing the tools to develop diagnostics that identify the patients most likely to benefit from molecularly targeted drugs.

Tumors of a primary site in many represent a heterogeneous collection of diseases that differ in pathophysiology, natural history, and sensitivity to treatment. These diseases differ with regard to the mutations that cause them and drive their invasion. The heterogeneous nature of tumors of the same primary site offers new challenges for drug development and clinical trial design. Physicians have always known that cancers of the same primary site were heterogeneous with regard to natural history and response to treatment. This understanding sometimes led to conflicts with statisticians over the use of subset analysis in the analysis of clinical trials. Although most statisticians expressed concern about the potential for false positive findings results from *post hoc* subset analysis, some practitioners rejected the results of clinical trials whose conclusions were based on average effects. Today we have better tools for characterizing the tumors biologically and using this characterization in the design and analysis of clinical trials that utilize this information prospectively.

Most oncology drugs are being developed for defined molecular targets. In some cases the targets are well understood and there is a compelling biological basis for restricting development to the subset of patients whose tumors are characterized by deregulation of the drug target. For other drugs there are multiple targets and more uncertainty about how to measure whether a drug target is driving tumor invasion in an individual patient (1). It is clear that the primary analysis of the new generation of oncology clinical trials must consist of more than just treating broad patient populations and testing the null hypothesis of no average effect. But it is also clear that the tradition of *post hoc* data dredging subset analysis is not an adequate basis for predictive oncology. For establishing practice standards and for drug approvals we need prospective analysis plans that provide for both preservation of the type I experiment-wise error rate and for focused predictive analyses that can be used to reliably select patients in clinical practice for use of the new regimen (2–4). These two primary objectives involve co-development of a drug and a companion diagnostic.

In the following sections we summarize some of the designs that are available for the co-development of a drug and companion diagnostic. Developing new treatments with companion diagnostics or predictive biomarkers for identifying the patients who benefit does not make drug development simpler, quicker, or cheaper as is sometimes claimed. Actually it makes drug development more complex and probably more expensive. But for many new oncology drugs it should increase the chance of success. It may also lead to more consistency in results among trials and increase the proportion of patients who benefit from the drugs they receive. This approach also has great potential value for controlling societal expenditures on health care.

The ideal approach to co-development of a drug and companion diagnostic involves: (i) identification of a predictive biomarker based on understanding the mechanism of action of the drug and the role of the drug target in the pathophysiology of the disease. This biological understanding should be validated and refined by pre-clinical studies and early phase clinical trials. The predictive biomarkers for successful cancer drugs have generally involved a single gene or protein rather than a multivariate classifier. Multivariate classifiers have been found some use as prognostic indicators that reflect a combination of the pace of the disease and the effect of standard therapy. Multivariate classifiers have rarely been used as predictive biomarkers for response to specific drugs because their use often reflects an incomplete understanding of the mechanism of action of the drug or the role of its molecular target. (ii) Development of an analytically validated test for measurement of that biomarker. Analytically validated means that the test accurately measures what it is supposed to measure, or if there is no gold-standard measurement, that the test is reproducible and robust. (iii) Use of that test to design and analyze a new clinical trial to evaluate the effectiveness of that drug and how the effectiveness relates to the biomarker value.

In the enrichment and stratified designs described below, biomarker discovery and determination of the threshold of positivity is performed prior to the phase III trial. Cancer biology is complex, however, and it is not always possible to have everything sorted out in this way before launching the phase III clinical trials. We will also discuss designs and prospective analysis plans that permit one to adaptively determine the best threshold of positivity for the biomarker and designs that incorporate multiple candidate biomarkers.

## Targeted (Enrichment) Designs

Designs in which eligibility is restricted to those patients considered most likely to benefit from the experimental drug are called “targeted designs” or “enrichment designs.” With an enrichment design, the analytically validated diagnostic test is used to restrict eligibility for a randomized clinical trial comparing a regimen containing a new drug to a control regimen. This approach has now been used for pivotal trials of many drugs whose molecular targets were well understood in the context of the disease. Several authors (5–9) studied the efficiency of this approach relative to the standard approach of randomizing all patients without using the biomarker test at all. The efficiency of the enrichment design depends on the prevalence of test positive patients and on the effectiveness of the new treatment in test negative patients. When fewer than half of the patients are test positive and the new treatment is relatively ineffective in test negative patients, the number of randomized patients required for an enrichment design is dramatically smaller than the number of randomized patients required for a standard design. For example, if the treatment is completely ineffective in test negative patients, then the ratio of number of patients required for randomization in the enrichment design relative to the number required for the standard design is approximately 1/γ^2^ where γ denotes the proportion of patients who are test positive. The treatment may have some effectiveness for test negative patients either because the assay is imperfect for measuring deregulation of the putative molecular target or because the drug has off-target anti-tumor effects. Even if the new treatment is half as effective in test negative patients as in test positive patients, however, the randomization ratio is approximately 4/(γ + 1)^2^. This equals about 2.56 when γ = 0.25, i.e., 25% of the patients are test positive, indicating that the enrichment design reduces the number of required patients to randomize by a factor of 2.56.

The enrichment design was very effective for the development of trastuzumab even though the test was imperfect and has subsequently been improved. Simon and Maitournam (5, 6) also compared the enrichment design to the standard design with regard to the number of screened patients. We have made the methods of sample size planning for the design of enrichment trials available on line at http://brb.nci.nih.gov. The web-based programs are available for binary and survival/disease-free survival endpoints. The planning takes into account the performance characteristics of the tests and specificity of the treatment effects. The programs provide comparisons to standard non-enrichment designs based on the number of randomized patients required and the number of patients needed for screening to obtain the required number of randomized patients.

The enrichment design is appropriate for contexts where there is a strong biological basis for believing that test negative patients will not benefit from the new drug. In such cases, including test negative patients may raise ethical concerns and may confuse the interpretation of the clinical trial. As described in the section on “stratification designs,” if test negative patients are to be included then one should ensure that a sufficient number of test positive patients are included to provide an adequately powered evaluation. Often this is not done and instead one sees a mixed population of patients in an inadequately sized trial leading to ambiguous conclusions.

The enrichment design does not provide data on the effectiveness of the new treatment compared to control for test negative patients. Consequently, unless there is compelling biological or phase II data that the new drug is not effective in test negative patients, the enrichment design may not be adequate to support approval of the test. If the biological rationale or phase II data is strong, however, then the test can be approved for identifying a subset of patients for whom an effective drug exists, rather than for distinguishing patients who do and do not benefit from the new drug.

In oncology, sequencing of tumor DNA to test for point or structural alterations in genes whose protein products are druggable is rapidly becoming part of the standard diagnostic workup at advanced cancer centers. Regulatory body approvals of drugs for populations defined by such tests will require that the tests be shown to have good analytical performance (10).

## Biomarker Stratified Design

When a predictive classifier has been developed but there is not compelling biological or phase II data that test negative patients do not benefit from the new treatment, it is generally best to include both classifier positive and classifier negative in the phase III clinical trials comparing the new treatment to the control regimen. In this case it is essential that an analysis plan be pre-defined in the protocol for how the predictive classifier will be used in the analysis. The analysis plan will generally define the testing strategy for evaluating the new treatment in the test positive patients, the test negative patients, and overall. The testing strategy must preserve the overall type I error of the trial and the trial must be sized to provide adequate statistical power for these tests. It is not sufficient to just stratify, i.e., balance, the randomization with regard to the classifier without specifying a complete analysis plan. The main value of “stratifying” (i.e., balancing) the randomization is that it assures that only patients with adequate test results will enter the trial. Pre-stratification of the randomization is not necessary for the validity of inferences to be made about treatment effects within the test positive or test negative subsets. If an analytically validated test is not available at the start of the trial but will be available by the time of analysis, then it may be preferable not to pre-stratify the randomization process.

The purpose of the pivotal trial is to evaluate the new treatment overall and in the subsets determined by the pre-specified classifier (generally biomarker plus cut-point for positivity). The purpose is not to modify or optimize the classifier unless an adaptive design is used. Several primary analysis plans have been described (10–12) and a web-based tool for sample size planning for some of these analysis plans is available at http://brb.nci.nih.gov For example, If one has moderate strength evidence that the treatment, if effective at all, is likely to be more effective in the test positive cases, one might first compare treatment versus control in test positive patients using a threshold of significance of 5%. Only if the treatment versus control comparison is significant at the 5% level in test positive patients, will the new treatment be compared to the control among test negative patients, again using a threshold of statistical significance of 5%. This sequential approach controls the overall type I error at 5%. To have 90% power in the test positive patients for detecting a 50% reduction in hazard for the new treatment versus control at a two-sided 5% significance level requires about 88 events of test positive patients. If at the time of analysis the event rates in the test positive and test negative strata are about equal, then when there are 88 events in the test positive patients, there will be about 88(1 − γ)/γ events in the test negative patients where γ denotes the proportion of test positive patients. If 25% of the patients are test positive, then there will be approximately 264 events in test negative patients. This will provide approximately 90% power for detecting a 33% reduction in hazard at a two-sided significance level of 5%. In this case, the trial will not be delayed compared to the enrichment design, but a large number of test negative patients will be randomized, treated, and followed on the study rather than excluded as for the enrichment design. This will be problematic if one does not, *a priori*, expect the new treatment to be effective for test negative patients. In this case it will be important to establish an interim monitoring plan to terminate accrual of test negative patients when interim results and prior evidence of lack of effectiveness makes it no longer viable to enter them.

In the situation where one has more limited confidence in the predictive marker it can be effectively used for a “fall-back” analysis. In Simon and Wang (13), we proposed an analysis plan in which the new treatment group is first compared to the control group overall. If that difference is not significant at a reduced significance level such as 0.03, then the new treatment is compared to the control group just for test positive patients. The latter comparison uses a threshold of significance of 0.02, or whatever portion of the traditional 0.05 not used by the initial test. If the trial is planned for having 90% power for detecting a uniform 33% reduction in overall hazard using a two-sided significance level of 0.03, then the overall analysis will take place when there are 297 events. If the test is positive in 25% of patients and the event rates in test positive and test negative patients are about equal at the time of analysis, then when there are 297 overall events there will be approximately 75 events among the test positive patients. If the overall test of treatment effect is not significant, then the subset test will have power 0.75 for detecting a 50% reduction in hazard at a two-sided 0.02 significance level. By delaying the treatment evaluation in the test positive patients power 0.80 can be achieved when there are 84 events and power 0.90 can be achieved when there are 109 events in the test positive subset. Wang et al. have shown that the power of this approach can be improved by taking into account the correlation between the overall significance test and the significance test comparing treatment groups in the subset of test positive patients (14). So if, for example a significance threshold of 0.03 has been used for the overall test, the significance threshold for used for the subset can be somewhat >0.02 and still have the overall chance of a false positive claim of any type limited to 5%. Real world experience with stratification and enrichment designs are described by Freidlin et al. (15) and by Mandreakar and Sargent (16). Freidlin et al. (17) describe a randomized phase II design for providing information for the design of the phase III trial in cases where there is not a strong biological rationale for the enrichment approach.

### Interim monitoring of test negative patients

Interim monitoring of outcome for the test negative patients is very important in clinical trials where there is preliminary evidence that efficacy of the new regimen may be limited to the test positive patients. One approach is to perform an interim analysis focused on the test negative patients using a standard futility monitoring statistical plan for the primary endpoint of the clinical trial. Such methods are usually either based on the standardized treatment effect or the conditional power of rejecting the null hypothesis at the end of the trial. One simple approach is to compute the standardized treatment effect in the test negative patients at a time when half of the events in test negative patients projected to occur by the end of the trial have occurred. If the treatment effect is going in the wrong direction, then accrual to the test negative stratum ceases. This type of futility analysis is designed to be conservative enough that the power at the end of the trial for detecting a treatment effect is minimally reduced. This type of futility monitoring is used in the design proposed by Wang et al. (14) but in many cases it provides very limited protection for test negative patients for use in biomarker driven designs. Depending on the accrual rate and survival distributions, by the time half of the primary endpoint events have occurred for the test negative patients, the accrual of test negative patients may be close to complete.

An alternative approach would be to base the futility monitoring of the test negative patients on an intermediate endpoint rather than on the primary endpoint of the trial. There would be no assumption that the intermediate endpoint is a true surrogate for the primary endpoint, only that if there is no treatment effect on the intermediate endpoint, then there is unlikely to be a treatment effect for the primary endpoint. With this limited assumption, made for most phase II trials, the futility analysis can be performed at an earlier time so that a finding of futility will limit the number of test negative patients accrued.

In Karuri and Simon (18) we introduced a phase III design for this setting in which futility monitoring of the test negative patients is performed based on a joint prior joint distribution for the treatment effects in test negative and test positive patients. That prior distribution enables the trial investigator to represent the prior evidence that treatment effect will be reduced for test negative patients and use that information in monitoring the clinical trial. Although the formulation is Bayesian, the rejection region based on posterior probability is calibrated so that type I errors satisfy the usual frequentist requirements.

## Biomarker Adaptive Threshold Design

In Jiang et al. (19) we reported on a “Biomarker Adaptive Threshold Design” for situations where a biomarker is available at the start of the trial, but a cut-point for converting the value to a binary classifier is not established. For example, this design could be used with a FISH assay for EGFR positivity without pre-specification of the threshold of positivity. Tumor specimens are collected from all patients at entry, but the value of the biomarker is not used as an eligibility criteria. Their analysis plan does not stipulate that the assay for measuring the index needs to be performed in real time. Two analysis plans were described. Analysis plan A begins with comparing outcomes for all patients receiving the new treatment to those for all control patients. If this difference in outcomes is significant at a pre-specified reduced significance level α_1_ (e.g., 0.03) then the new treatment is considered effective for the eligible population as a whole. Otherwise, a second stage test is performed using significance threshold α_2_ = 0.05 − α_1_. The second stage test involves finding the cut-point s* for the biomarker score which leads to the largest treatment effect in comparing *T* to *C* restricted to patients with score greater than s*. Jiang et al. employed a log-likelihood measure of treatment effect and let L* denote the log-likelihood of treatment effect when restricted to patients with biomarker level above s*. The null distribution of L* was determined by repeating the analysis after permuting the treatment and control labels a thousand or more times, recomputing s* and L* each time. If the permutation statistical significance of L* is <0.05 − α_1_ (e.g., 0.02), then treatment *T* is considered superior to *C* for the subset of the patients with biomarker level above s*.

The advantage of procedure A is its simplicity and that it explicitly separates the test of treatment effect in the broad population from the subset selection. However, the procedure takes a conservative approach in adjusting for multiplicity of combining the overall and subset tests. An alternative analysis plan B proposed by Jiang et al. does not use a first stage comparison of treatment groups overall. Consequently, plan B is more appropriate to settings in which there is greater expectation that treatment effect will be limited to a marker defined subset. With analysis plan B they determine the cut-point value *b* at which *w*(*b*)*S*(*b*) is maximized, where *w*(*b*) is a pre-defined weight function. The weight function is used to give greater emphasis to the *b* = 0 subset, that is, the subset containing all patients (marker value is initially normalized to the 0–1 interval). Let *T*(*b*) = *w*(*b*)*S*(*b*) denote the value of the maximized weighted partial log-likelihood. The statistical significance of *T*(*b*) is determined by generating the null distribution by repeating the optimization procedure for many cases of randomly permuted data. With either procedure A or B, a confidence interval for the optimal cut-point *b* is generated by bootstrap re-sampling of the maximum likelihood estimate of the cut-point based on a proportional hazards model with an unknown cut-point and an unknown treatment effect for patients with biomarker values above the cut-point. Since the treatment is presumed effective only for patients with biomarker above the threshold *b*, the confidence coefficient associated with a given biomarker value *x* can be interpreted as the probability that a patient with marker value *x* benefits from the new treatment.

In Jiang et al. (19) we also provided an approach to sample size planning for the biomarker adaptive threshold design. With analysis strategy A, sample size is determined in the traditional manner for overall comparison of the treatment arms but powering the trial for using a reduced significance level *a*_1_, e.g., 0.03. With analysis plan B a larger sample size is used to provides good power for establishing the statistical significance of treatment effects restricted to patients with biomarker values above an initially unknown cut-point.

## Adaptive Enrichment Designs

The adaptive threshold design described above (19) enables one to conduct the phase III clinical trial without pre-specifying the cut-point for the biomarker. It provides for a valid statistical significance test that has good statistical power against alternative hypotheses that the treatment effect is limited to patients with biomarker values above some unknown level, and it provides a confidence interval for estimation of the cut-point. These analyses are, however, performed at the end of the trial and accrual during the trial is not restricted by biomarker value. In Simon and Simon (20), we introduced a very general class of adaptive enrichment designs in which the eligibility criteria are adaptively adjusted during the course of the trial in order to exclude patient subsets unlikely to benefit from the new regimen. Others have also studied adaptive enrichment designs (21–23). Wang et al. (21) and Simon and Simon (20) provide general frameworks for adaption and identify statistical significance tests that provide protection of the study-wise type I error under broad conditions. In Simon and Simon (20) we applied this framework to the setting of adaptive threshold enrichment of a single biomarker.

## Designs That Evaluate a Small Number of Biomarkers

Because of the complexity of cancer biology, there are many cases in which the biology of the target is not sufficiently well understood at the time that the phase III trials are initiated to restrict attention to a single predictive biomarker. The analysis plan used in the adaptive threshold design (19) is based on computing a global test based on a maximum test statistic. For the adaptive threshold design, the maximum is taken over the set of cut-points of a biomarker score. The idea of using a global maximum test statistic is much more broadly applicable, however. For example, suppose multiple candidate binary tests, *B*_1_, …, *B_K_* are available at the start of the trial. These tests may or may not be correlated with each other. Let *L_k_* denote the log-likelihood of treatment effect for comparing *T* to *C* when restricted to patients positive for biomarker *k*. Let L* denote the largest of these values and let k* denote the test for which the maximum is achieved. As for the adaptive threshold design, the null distribution of L* can be determined by repeating the analysis after permuting the treatment and control labels a thousand or more times. If the permutation statistical significance of L* is <0.05 − α_1_ (e.g., 0.02), then treatment *T* is considered superior to *C* for the subset of the patients positive for biomarker test k*. The stability of the indicated set of patients who benefit from *T* (i.e., k*) can be evaluated by repeating the computation of k* for bootstrap samples of patients. This approach can be useful when the number of candidate biomarkers is small, as it should be by the time a phase III trial is initiated. Some of the adaptive enrichment designs (20) can also be employed in that setting with multiple biomarker candidates with or without known cut-points of positivity.

## Adaptive Classification Based on Screening Candidate Biomarkers

Designs such as the “adaptive signature design” have been developed for adaptive multivariate classifier development and internal validation based on high dimensional genomic tumor characterization (24). This design employs a “learn and confirm” structure in which a portion of the patients are used to select the biomarker hypothesis, i.e., to develop an “indication classifier” which identifies the target population of patients in which the test treatment is most likely to be effective, and to use the remainder of the patients to test the treatment effect in that subset. The adaptive signature design does not modify eligibility criteria. It is adaptive in the sense that the treatment effect is tested in a single subset determined based on the clinical trial data but in a manner that separates classifier development from testing of treatment effect. This is dramatically different than the current practice of *ad hoc* analysis in multiple subsets with no control of type I error or in using the full dataset to both develop a classifier and to classify patients for purpose of hypothesis testing. Since the adaptive signature design does not use the patients on which the classifier was developed for the testing of the treatment effect, it thus avoids the inflation of type I error described by Wang et al. (25) for other approaches. Scher et al. described the use of the adaptive signature design for planning a pivotal trial in advanced prostate cancer (26). The key idea of the adaptive signature approach is to replace multiple significance testing based subset analysis with development and internal validation of a single “indication classifier” that informs treatment selection for individual patients based on their entire vector of covariate values.

The adaptive signature design approach is very general with regard to the methodology applied to the training set for identifying the single candidate subset in which treatment effect will be tested in the validation set. In many cases this can be accomplished by developing a model for predicting outcome as a function of treatment, selected biomarkers and treatment by biomarker interactions. In the original adaptive signature design paper this was accomplished by screening all the candidate biomarkers using predictive models that include the main effect of treatment, main effect of a single biomarker, and the corresponding interaction of that biomarker with treatment. Candidate markers which exhibited an interaction nominally significant at a pre-specified level were included in a final multivariate predictive model. A machine learning weighted voting model was used in the original paper to classify patients as either likely to benefit from the new treatment or not likely to benefit from the new treatment. The tuning parameters for this classifier were optimized by cross-validation in the training set. The multivariate model was then used to classify the patients in the validation set, and the treatment effect was evaluated in the subset of the patients in the validation set that were classified as likely to benefit from the new treatment based on the classifier developed in the training set.

Many other methods of classifier development can be employed using the training set. It is important to recognize, however, that one is not developing a prognostic classifier. The classifier is used to classify patients as likely to benefit from the new treatment. One could develop prognostic classifiers separately for the treatment and control groups using standard penalized regression methods and then classify patients based on which prognostic classifier predicts the better outcome. More commonly, however, single predictive models have been used based on screening candidate markers based on their univariate interaction with treatment. Matsui et al. (27) used their model to predict a continuous score reflecting the expected benefit for the new treatment relative to the control rather than just classifying patients into one of two subsets. Gu et al. (28) have developed a two-step strategy for developing a model for predicting outcome as a function of treatment and selected biomarkers. The biomarkers are selected using a group lasso approach in which the main effects of a biomarker are grouped with the interactions of that marker with treatments and can be used with two or more treatments.

Freidlin et al. (29) described further extensions of the adaptive signature approach. They use cross-validation to replace sample splitting of the trial into a training set and test set in order to increase the statistical power.

## Conclusion

Recognition of the molecular heterogeneity of human diseases such as cancers of a primary site and the tools for characterizing this heterogeneity presents new opportunities for the development of more effective treatments and challenges for the design and analysis of clinical trials. In oncology, treatment of broad populations with regimens that do not benefit most patients is less economically sustainable with expensive molecularly targeted therapeutics and less likely to be successful. The established molecular heterogeneity of human diseases requires the development of new approaches to use randomized clinical trials to provide a reliable basis predictive medicine (3, 4). This paper has attempted to review here some prospective phase III designs for the co-development of new therapeutics with companion diagnostics.

## Conflict of Interest Statement

The author declares that the research was conducted in the absence of any commercial or financial relationships that could be construed as a potential conflict of interest.
